# Integrated diagnostic approach for Leptospira spp. detection in SMEDI associated reproductive disease of swine

**DOI:** 10.1016/j.vas.2026.100700

**Published:** 2026-05-16

**Authors:** Matthias Eddicks, Annika Seifert, Julia Gründl, Katrin Strutzberg-Minder, Lina Eddicks, Robert Tabeling, Mathias Ritzmann

**Affiliations:** a Clinic for Swine at the Centre for Clinical Veterinary Medicine of the Ludwig-Maximilians-Universität München, 85764 Oberschleissheim, Germany; b IVD Innovative Veterinary Diagnostics (IVD GmbH), Consiliary Laboratory of the German Veterinary Medical Society (GVMS) for Leptospira spp., 30926 Seelze, Germany; c Institute of Veterinary Pathology at the Centre for Clinical Veterinary Medicine of the Ludwig-Maximilians-Universität München, 80539 München, Germany; d MSD Animal Health, Intervet Deutschland GmbH, 85716 Unterschleissheim, Germany

**Keywords:** Diagnostic, Swine, Leptospirosis, qPCR, Meconium, Stomach-content

## Abstract

Leptospirosis is a notifiable disease in human and all mammals in Germany. Besides late term abortions, leptospiral infection of sows is also mentioned as a differential in terms of SMEDI. The objective of the present study was to apply an integrated diagnostic approach in such cases under field conditions and to evaluate best suited specimen for molecular detection of *Leptospira* spp. in affected fetuses. Therefore, MAT of fetal and sow sera and LipL32 and 16S RNA gene qPCR for fetal specimens (kidney, liver, stomach content, meconium) was used for *Leptospira* spp. diagnostic to detect pathogenic serovars and to distinguish between pathogenic groups I and II of *Leptospira* spp. in SMEDI associated cases, respectively. In total, 36 litters with 142 fetuses from 16 farms were enrolled in this examination. In total 44.4 % of the sow sera were positive by MAT. Six out of sixteen (37.5 %) serum samples were positive for only one serovar, whereas 62.5 % (10/16) showed multiple positive results. MAT positivity for serovars Bratislava, Pomona, Australis and Autumnalis in sows was significantly associated with positive qPCR results from the corresponding offspring. All PCR positive samples (n = 20) revealed that leptospires belonged to subclade P1 or pathogenic group I identified by 16S RNA gene PCR. Highest detection rates and DNA loads were present in kidney samples whereas stomach and meconium samples revealed the lowest rates of detection and DNA loads.

## Introduction

Leptospirosis is known as the most widespread zoonosis worldwide (Adler & de la Pena Moctezuma, 2010) and displays a notifiable disease in humans and all mammals in Germany. Pigs are susceptible to infection with a broad range of pathogenic *Leptospira* serovars. However, they are regarded as maintenance hosts for the serogroups Pomona and Australis, while serogroups Icterohaemorrhagiae, Grippotyphosa, and Tarassovi are more frequently associated with incidental infections in swine (Arent & Ellis, 2019).

Based on the results of a passive surveillance from the consiliary laboratory for Leptospirosis in Germany, 59.3 % - 78.2 % of farms and 16.3 % - 30.9 % of single sera from swine, sent in for diagnostic approaches (monitoring or case-related submissions) from 2011 to 2016 were positive in the microscopic agglutination test (MAT) (Strutzberg-Minder et al., 2018). Recently published data reported a global seroprevalence estimation for *Leptospira* spp. in swine of 21.9 %, and 13.3 % for Europe. However, even higher seroprevalence in swine were calculated for South America (36.4 %), North America (34.0 %), Africa (22.1 %) or Oceania (17.4 %) (Gomes de Araujo et al., 2023). Referring to single sera, data from Germany indicate a seroprevalence of 20.2 % with serovar Bratislava (11.6 %) as the most prevalent one in the German pig population, followed by the serovars Australis with a seropositivity of 7.3 %, Icterohaemorrhagiae with 4.0 %, Copenhageni with 4.0 %, Autumnalis with 3.7 %, Canicola with 2.0 % and Pomona with 1.2 % (Strutzberg-Minder et al., 2018). More recent data from France report a *Leptospira* seroprevalence of 22.7 % in pigs with Australis (48.5 %) and Icterohaemorrhagiae (38.2 %) identified as the most prevalent ones. Moreover, serovars Autumnalis (6.1 %), Panama (5.0 %), Ballum (1.2 %), Tarassovi (0.5 %), Sejroe (0.2 %), Grippotyphosa (0.2 %), Bataviae (0.1 %), and Pomona (< 0.1 %) were also detected (Naudet et al., 2022).

Leptospiral infections occur regularly on swine farms but endemic infection status does not necessarily result in clinical outbreaks of leptospirosis. However, initial infections in a herd or a reduced herd immunity may result in clinically apparent infections (Arent & Ellis, 2019). As leptospires can persist in the kidneys and are shed in urine, subclinically infected carrier pigs represent an important reservoir for the further spread of the bacteria (Fish et al., 1963). In case of clinical outbreaks, reproductive disorders including infertility, fetal mummification, abortions, stillborn or weak-born fetuses or pigs (Arent & Ellis, 2019) are notable changes. SMEDI is also mentioned (Althouse et al., 2019; Eddicks et al., 2023) as a consequence of leptospiral infection in pigs. The appearance of SMEDI litters depends on the time of infection of the fetuses. The characteristic appearance of these litters (reduced litter size, mummified, macerated or stillborn fetuses in ascending size within one litter) is explained by initial sow viremia-associated infection of fetuses and subsequent horizontal spread of the pathogen within the uterus (Truyen & Streck, 2019; Zeeuw et al., 2007). Horizontal spread of leptospires was also assumed in consequence of the results of a controlled infection of sows with serovar Pomona and Sejroe (Fennestad & Borg-Petersen, 1966). Moreover, maceration of fetuses as a sign of bacterial decomposition (Lefebvre, 2015) displays a typical finding (Eddicks et al., 2023; Fennestad & Borg-Petersen, 1966). In terms of reproductive clinical signs, MAT of serum samples from affected sows, or PCR examinations of tissue samples from dead born fetuses are routinely performed laboratory examinations for an etiological diagnostic approach (Bolin, 1994). The MAT is seen as the standard serological test for *Leptospira* spp. diagnostic for individual animals or prevalence studies (World Organisation for Animal Health, 2024) but the use of vaccines and the wide distribution of *Leptospira* spp. in the domestic pig population with subclinical course of the infection, exacerbates the interpretation of the results. Moreover, although the sensitivity of MAT is good, serological cross-reactions between serovars and serogroups appear and one infected animal is likely to have antibodies against other serovars, mostly at lower levels (World Organisation for Animal Health, 2024). The isolation of *Leptospira* spp. from tissues seems to be the definitive method of diagnosis (Bolin, 1994; Bolin et al., 1991). However, the restricted availability of this method, because of the difficulties to grow *Leptospira* spp., and thus, the long diagnostic time, especially in the field settings (Bolin, 1994), display major limitations. From this point of view, PCR seems to be a promising approach to overcome these drawbacks (Arent & Ellis, 2019). Meanwhile PCRs have become available that are able to detect pathogenic serovars (Ferreira et al., 2014) based on detection of LipL32 gene and even distinguish between pathogenic groups I and II based on 16S RNA gene of *Leptospira* spp. (Perez et al., 2020), recently reclassified by Vincent et al. (2019) as P1 and P2.

Thoughtful sample submission in terms of abortions or stillbirth may include lung, liver and kidney from affected fetuses (Bolin, 1994), moreover stomach content is suggested as an adequate sample in cases of suspected bacterial abortion e.g. Brucellosis (Althouse et al., 2019). As meconium contains digested amnion fluid (Harries, 1978) it may display an easily gainable diagnostic specimen. Concerning *Leptospira* spp. PCR or IHC and FAT on fetal kidney and dam serology is suggested as diagnostic approach (Althouse et al., 2019). Moreover, the detection of antibodies against *Leptospira* spp. in stillborn fetuses, if infected after the 70th day of gestation (Arent & Ellis, 2019; Pensaert et al., 2004), is a diagnostic possibility (Arent & Ellis, 2019). The detection of antibodies in fetal serum is considered diagnostic of leptospiral abortion (Arent & Ellis, 2019). Not all specimens mentioned are always available for further diagnostic examinations or tested in terms of SMEDI under field conditions. Thus, the etiological diagnosis of *Leptospira* spp. infections remains a challenge in such cases. The objective of the present study was to apply an integrated diagnostic approach in such cases under field conditions and to evaluate best suited specimen for molecular detection of *Leptospira* spp. in affected fetuses under field conditions. Therefore, we included established specimens suggested for leptospiral diagnostic and added easily gainable material (meconium) with the aim of enabling veterinarians to optimize leptospiral diagnostics in such cases. The examinations included serological examinations (MAT) of sow sera, fetal heart blood and molecular biological examinations of tissue pools, stomach content and meconium from corresponding dead fetuses were carried out by MAT or PCR, respectively.

## Material and methods

### Study population and laboratory diagnostics

Diagnostic specimens for this study originate from a SMEDI database from diagnostic transmittals collected between July 2021 and February 2022. Within this database, tissue samples from 142 fetuses of 36 SMEDI litters from 16 farms, of which serum samples (n = 36) from the corresponding sows were also available, were enrolled in this examination.

All laboratory examinations were conducted in the consiliary laboratory for *Leptospira* spp. in Germany (Innovative Veterinary Diagnostics (IVD GmbH), Seelze, Germany). All samples were collected in the Institute of Veterinary Pathology at the Centre for Clinical Veterinary Medicine of the Ludwig-Maximilians-Universität München.

### Sample collection for the database

Serum from the sows and litters originate from field cases of SMEDI of different federal states of Germany and were sent frozen to our facility for diagnostic purposes. Only whole litters (all dead borne fetuses of an affected litter) with typical SMEDI appearance were included in this study. Photographic documentation of all litters was done after thawing at room temperature and the crown-rump length, bodyweight, phenotype (mummified, macerated, stillborn and, or if lung swim test was positive, weak-born) of each fetus were documented. Subsequently, four fetuses per litter were selected by systematic random sampling. To ensure that the sampled population reflected the overall phenotype distribution and size variability of the study population, a systematic sampling approach with a fixed interval was applied. The sampling interval was determined by dividing the number of fetuses per litter by the target sample size (four fetuses per litter), resulting in larger intervals for larger litters and thereby capturing both size and phenotypic variation.

Rather than selecting a purely random starting point, the starting position was systematically shifted by one fetus across four consecutive litters and reset to the first fetus in every fifth litter. This approach was chosen to reduce potential selection bias and to better account for heterogeneity within and between litters.

In litters with four or fewer fetuses (including two litters with only three fetuses), all available fetuses were included. In total, 142 fetuses from 16 litters were examined. Table 1 summarizes the total SMEDI study population, the subset meeting the inclusion criteria, and the final study population, including phenotype distribution.

**Table 1:** Percentage comparison of fetal phenotypes among all documented fetuses (total population), the population meeting the inclusion criteria, and the randomly selected study population.

Phenotype	Total population (n = 358 fetuses)	Population fulfilling inclusion criteria (n = 327 fetuses)	Randomly sampled population (n = 142 fetuses)
Mummified	30.4 %	27.8 %	26.8 %
Macerated	21.5 %	22.3 %	21.8 %
Stillborn	44.7 %	46.5 %	43.7 %
Weak-born	3.4 %	3.4 %	7.7 %
Total	100 %	100 %	100 %

**Fig. 1:**
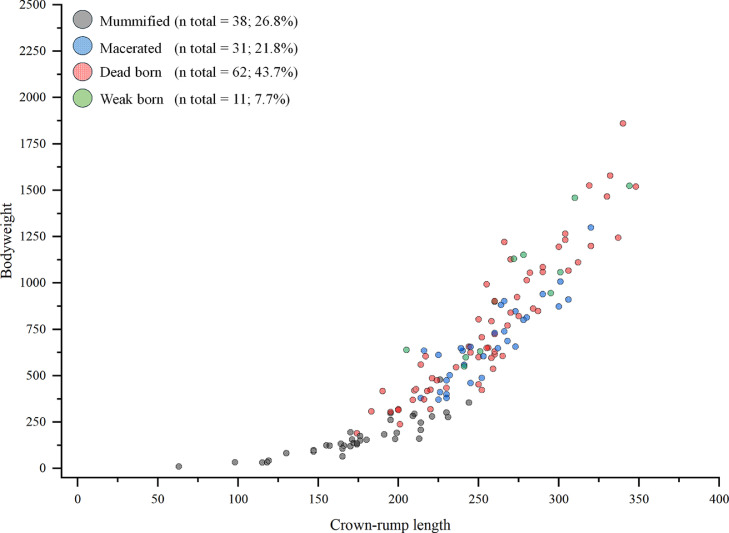
Crown-rump length and bodyweight as well as number and percentage of all enrolled fetuses (n = 142) by phenotype.

Fig. 1 displays more details concerning the number, phenotypes and size of all sampled fetuses.

From each fetus (n = 142), kidney, liver, stomach content and meconium were collected. Additionally, cardiac blood was aseptically collected from the fetuses using a sterile syringe and needle by puncture of the heart ventricles.

### Laboratory diagnostic examinations

Serum of the sows (n = 36) and heart blood from fetuses with an approximate age of ≥ 70 days (n = 100, based on their crown-rump-length (Evans & Sack, 1973)) were examined for antibodies against *Leptospira* spp. by MAT in accordance with the standards of the WOAH (World Organisation for Animal Health, 2024).

Therefore, live antigens of Leptospira serovars Australis (strain Ballico), Bratislava (strain Jez Bratislava), Canicola, (strain Hond Utrecht IV), Grippotyphosa (strain MoskvaV), Copenhageni (strain M20), Icterohaemorrhagiae(strain RGA), Pomona (strain Pomona), Hardjo (strain Hardjoprajitno), Saxkoebing (strain Mus 24), and Tarassovi (strain Perepelitsin) were used. All strains were supplied by the Leptospirosis Reference Laboratory (at KIT Biomedical Research, The Netherlands).

Sera were pretested at the final dilution of 1/100. Sera, with 50 % agglutination were retested to determine an endpoint using dilutions of sera beginning at 1/25 through 1/3200. Serum samples with the widely accepted minimum significant titer of 100 (reciprocal of the final dilution of serum with 50 % agglutination) were assessed positive. Fetal serum samples were pretested at the final dilution of 1:12,5 (World Organisation for Animal Health, 2024).

From each fetus (n = 142), kidney, liver, stomach content and meconium were pooled for molecular biological examinations. In case a pool was positive, all kinds of sample material were subsequently examined the same way individually. Pooled samples were examined by a multiplex real-time PCR, based on LipL32 gene (Ferreira et al., 2014) for detecting DNA from pathogenic *Leptospira,* and based on 16S RNA gene (Perez et al., 2020) for the identification of the newly differentiated subclades. The Mag-MAX™ Pathogen RNA/DNA kit, Applied Biosystems, was used for DNA isolation according to the manufacturer’s protocol. The result was rated as positive when the Ct-value of the PCR was < 40.

### Statistical examinations

Data processing was performed using Microsoft Excel 365 and statistical analyses were conducted using IBM SPSS Statistics version 28.0.1.0 for Microsoft® Windows. Laboratory test results were documented as metric scale (PCR Ct-values) and MAT log2-titer or nominal data (positive/negative). Moreover, epidemiological data including the parity of the sows, crown-rump-length (mm) and body weight (g) of the fetuses and their phenotypical appearance (mummified, stillborn macerated, stillborn fresh, weak-born) were documented the same way. Additionally, information about the vaccination scheme of the farms were included in the final analysis. Nominal data was tested for associations by Chi^2^ test and Odds ratio. Metric data was tested for normal distribution and either tested for associations or correlations by metric or parametric tests. Generalized estimating equations (GEE) with farm as subject and fetus as inner subject variable was calculated to estimate associations between the phenotype of fetuses and the detection rate of leptospiral DNA in the samples.

## Results

### MAT results

Antibodies against *Leptospira* spp. were present in 62.5 % (10/16) of the farms. In total 44.4 % (16/36; 95 % CI: 27.8 % - 61.1 %) of the sow sera were positive by MAT. The results concerning the individual serovars are shown in Table 1. Six out of sixteen (37.5 %) serum samples were positive for only one serovar whereas 62.5 % (10/16) showed multiple positive results (Table 2). Antibodies against *Leptospira* serovars Grippotyphosa, Hardjo, Saxkoebing and Tarassovi were not detected by MAT. MAT positive results for *Leptospira* serovar Canicola were only present in farm 6, which had vaccinated against *Leptospira* spp. Under consideration of LipL32 PCR positive or negative litters, the detection rate of antibodies against certain serovars was significantly higher for serovar Bratislava, Pomona and Autumnalis in corresponding sows (for details please refer to Table 2).

**Table 2:** Percentage of *Leptospira* spp. serovars of sow sera (n = 36) in descending order under consideration of LipL32 PCR positive or negative litters and Chi^2^ results (Fisher exact test) with Odds ratio and corresponding confidence interval in case of significance.

Serogroup	Serovar sow	Total MAT positive % (n)	MAT positive sows of PCR positive litter % (n)	MAT positive sows of PCR negative litter % (n)	*p*-value Odds ratio (CI)
Australis	Bratislava	38.9 % (14/36)	83.3 % (5/6)	30.0 % (9/30)	***p* = 0.024** OR:11.66 (1.18–114.59)
	Australis	16.7 % (6/36)	50 % (3/6)	10.0 % (3/30)	***p* = 0.045** OR: 9.00 (1.22–66.23)
Pomona	Pomona	19.4 % (7/36)	83.3 % (5/6)	6.7 % (2/30)	***p* < 0.001** OR: 70.00 (5.29–925.82)
Icterohaemorrhagiae	Icterohaemorrhagiae	16.7 % (6/36)	33.3 % (2/6)	13.3 % (4/30)	*P* = 0.256
	Copenhageni	16.7 % (6/36)	33.3 % (2/6)	13.3 % (4/30)	*p* = 0.256
Autumnalis	Autumnalis	13.9 % (5/36)	66.7 % (4/6)	3.3 % (1/30)	***p* = 0.001** OR: 58.00 (4.23–795.24)
Canicola	Canicola	5.6 % (2/36)	0.0 % (0/6)	6.7 % (2/30)	*p* = 1.00

In total only 23/100 (23.0 %) sera gained from heart blood samples of the fetuses with an estimated age > 70 days could be examined by MAT, because all other samples were hemolytic and thus, not suitable for an agglutination testing technique. Of these, seven (30.4 %) were from *Leptospira* spp. PCR positive fetuses, but all heart blood sera from the fetuses tested negative by MAT.

Titer highs in sow sera ranged from 100 up to 3200 of which the highest titers were from sows with *Leptospira* spp. PCR positive litters or sows with vaccination against *Leptospira* spp. The titers against all tested serovars from all positive sows are shown in Table 3. Titers from sows with PCR positive litters are tagged in orange, the overall highest titers for each sow are tagged in green. Farm 6 used a vaccine against *Leptospira* spp. for sows.

**Table 3:** MAT results from individual sows that tested positive for different serogroups and serovars. The highest titer for each sow is tagged in bolt. Litters from sows from farm 3 (3.1) and farm 10 (10.1, 10.2, 10.3, and 10.5) were *Leptospira* spp. PCR positive. Farm 6 used a vaccination against *Leptospira* (Porcilis® Ery+Parvo+Lepto, MSD Animal Health, Boxmeer, NL) for sows.

		Serogroup and serovar							
Farm	Sow	Australis			Autumnalis	Canicola	Icterohaemorrhagiae		Pomona
		Australis		Bratislava	Autumnalis	Canicola	Copenhageni	Ictero-haemorrhagiae	Pomona
2	2.1	-	**100**		-	-	-	-	-
3	3.1	100	1600		200	-	-	-	**3200**
4	4.2	-	-		-	-	**400**	200	-
	4.3	-	**100**		-	-	**100**	**100**	-
5	5.1	-	**100**		-	-	-	-	-
6	6.1	200	400		100	200	**3200**	**3200**	-
	6.2	200	**800**		-	100	200	400	100
7	7.1	-	**800**		-	-	-	-	-
10	10.1	-	200		-	-	-	-	**1600**
	10.2	-	400		100	-	100	-	**3200**
	10.3	200	400		400	-	-	100	**3200**
	10.5	400	**1600**		200	-	800	400	**1600**
14	14.1	-	**100**		-	-	-	-	-
15	15.1	-	-		-	-	-	-	**200**
16	16.1	-	**200**		-	-	-	-	-
	16.3	100	**200**		-	-	-	-	-

**Fig. 2:**
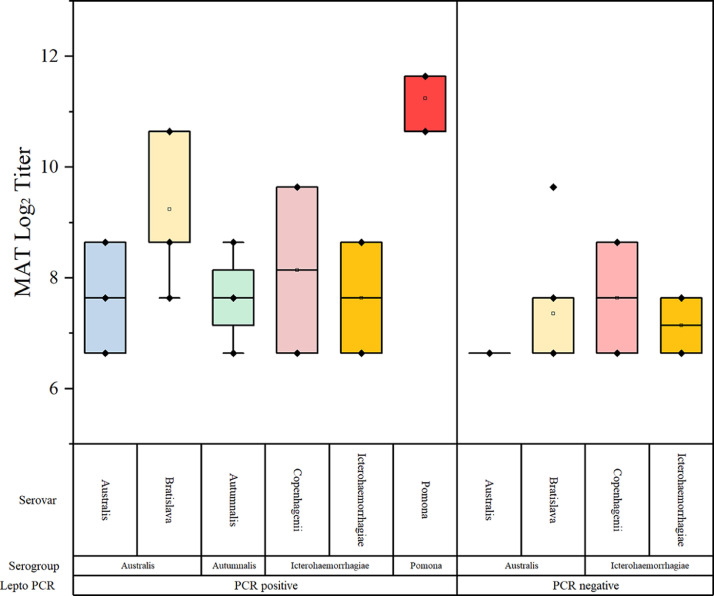
Boxplot of log_2_ transformed MAT titers in sera of sows with *Leptospira* spp. PCR positive or negative litters including median values. Only farms without *Leptospira* spp. vaccination included.

Under consideration of the PCR result of the corresponding litters, antibodies against *Leptospira* serovar Autumnalis were only present in sera with PCR positive litters or vaccination against *Leptospira* spp. Log_2_ transformed MAT titers of sow sera did not differ significantly within the groups PCR positive / negative. Log_2_ transformed MAT titers of sows excluding farm 6 (this farm used *Leptospira* vaccination) under consideration of the PCR results of the corresponding litters are shown in Fig. 2.

### Fetuses

**Fig. 3:**
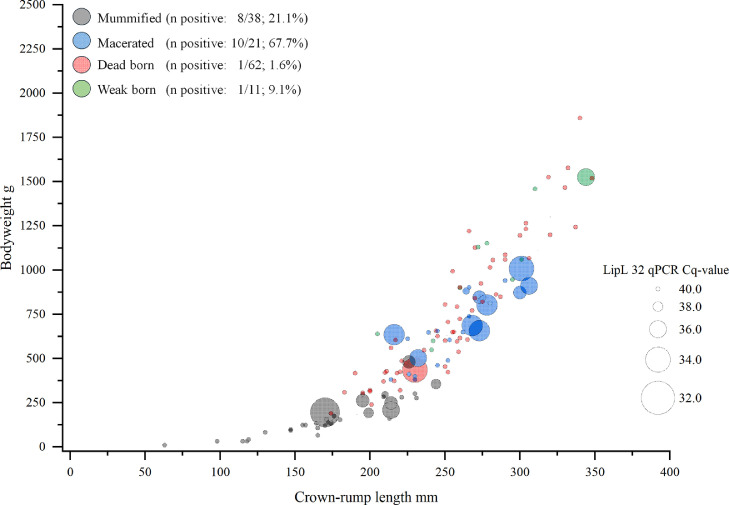
Bubble plot including the phenotype by color with number and % of LipL32 PCR positives, the crown-rump length and bodyweight of the fetuses and the DNA load by size of the bubbles.

Screening by qPCR for the pathogenic *Leptospira*-specific gene *lipL32* (LipL32 is the product (protein) of the gene) in pooled samples of single piglets revealed a total of 14.1 % (20/142) positive piglets, 16.6 % (6/36) positive litters and 12.5 % (2/16) positive farms. All of them belonged to subclade P1 or pathogenic group I identified by 16S RNA gene PCR. The detection rate but not the DNA load was significantly associated with the phenotypic appearance. Generalized estimating equations (GEE) model with farm as subject and fetuses as inner subject variable revealed that tissue pools from macerated fetuses were significantly more often leptospiral DNA positive compared to the other phenotypes (*p* < 0.001). The Odds to detect leptospiral DNA in tissue pools from macerated fetuses was 4.81 (95 % CI: 1.77 –13.00). Moreover, no significant correlation between the Ct-value and the crown-rump length and no significant association between the detection of leptospiral DNA and the crown-rump length was present. For more details, please refer to Fig. 3.

Specimens from PCR positive pools were subsequently examined individually. Highest rates of DNA detection were present in kidney samples whereas stomach content and meconium samples revealed the lowest rates of detection. In Table 4 the overall positive pools and the number and percentage of positive samples by examined tissue of all positive pools are shown.

**Table 4:** Amount of PCR positive specimens in all PCR positive pooled samples.

	Total PCR positive (16S + Lipl32)	PCR positive	N positives (% positives)
Pools	20 (100 %)	16S	18/20 (90.0 %)
		Lipl32	20/20 (100 %)
Kidney	19/20 (95.0 %)	16S	18/19 (94.7 %)
		Lipl32	19/19 (100.0 %)
Liver	17/20 (85.0 %)	16S	16/17 (94.1 %)
		Lipl32	17/17 (100.0 %)
Stomach content	16/20 (80.0 %)	16S	14/16 (87.5 %)
		Lipl32	12/16 (75.0 %)
Meconium	15/20 (75.0 %)	16S	11/15 (73.3 %)
		Lipl32	14/15 (93.3 %)

**Fig. 4:**
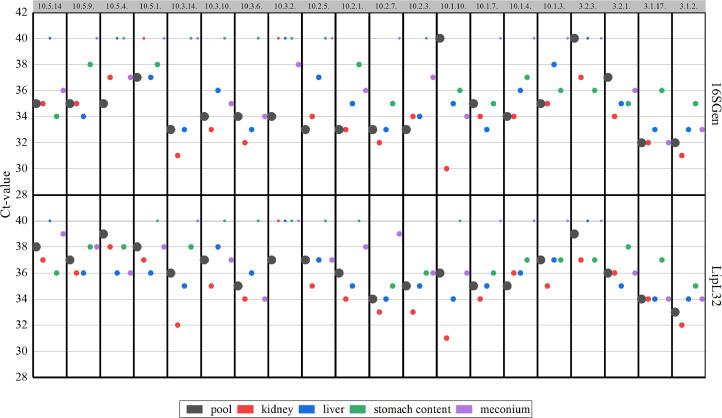
Trellis plot showing all qPCR-positive pools (LipL32 PCR or 16S rRNA gene PCR), each consisting of kidney, liver, stomach content, and meconium samples, along with the individual qPCR results (LipL32 PCR or 16S rRNA gene PCR) for each specimen within the respective pool. The upper x-axis indicates the pool ID, the lower x-axis represents the individual specimens, the left y-axis shows the Ct values, and the right y-axis indicates the PCR type used. Ct-values below 40 are rated as positive.

Besides being the most often positive sample type, kidney samples also contained the highest amount of *Leptospira* spp. DNA. The comparison of the Ct-values by pairwise comparison with Bonferroni-correction for multiple tests revealed significant lower Ct-values in kidney samples compared to stomach content (*p* = 0.001) for the 16S RNA gene PCR, and kidney (*p* = 0.002) or liver samples (*p* = 0.048) compared to stomach content for the LipL32 PCR. Under consideration of the used PCR, related samples Wilcoxon signed rank test revealed that for each tissue 16S RNA gene PCR had lower Ct-values compared to LipL32 PCR. As shown in Fig. 4, all qPCR-positive pools and their corresponding individual specimen results are displayed, illustrating the distribution of Ct values and the PCR methods used across pools and samples.

## Discussion

Leptospiral infections are regularly mentioned as a differential or cause of abortion, stillbirth or SMEDI (Arent & Ellis, 2019; Bolin et al., 1991; Saravi et al., 1989). In the present study, we focused on the indirect and direct detection of leptospiral infection associated with SMEDI in sows and their corresponding fetuses, using MAT and PCR, respectively. Due to this approach, the findings of this study do not reflect the overall prevalence of leptospiral infection in the pig population in Germany. Furthermore, given the limited number of available samples, the statistical results should be interpreted with caution. Nevertheless, the results indicate that *Leptospira* spp. should be considered in routine diagnostic investigations in such cases.

Based on MAT test, 62.5 % of the SMEDI affected farms and 44.0 % of the single sows were rated as positive for antibodies against *Leptospira* spp. by MAT within the present study. The percentage of seropositive farms is in line with passive surveillance findings of Strutzberg-Minder et al. (2018) from Germany, where 59.3 % - 78.3 % of farms were positive by MAT. Concerning the MAT results of single sera in our study, a higher rate of detection of anti-*Leptospira* spp. antibody positive sows (44 %) were recognized compared to the beforementioned study (16.3 % - 30.9 % positive sera). This difference might be ascribable to the difference of the study population. Whereas Strutzberg-Minder et al. (2018) conducted a passive surveillance in German farms with non-defined reproductive problems, we concretely examined material from clinical cases of SMEDI, which at least might explain a higher seroprevalence in the corresponding sow population as only verifiable affected sows were sampled. Moreover, differences concerning the percentage of multiple seropositive sow sera were also obvious (62.5 % in our study compared to 42.7 % from Strutzberg-Minder et al. (2018)). Admittedly, serovar Bratislava was the most often detected one which is comparable to the before mentioned results from Germany, however, serovar Pomona was detected markedly more often in our study compared to the German data from 2011 - 2016. Antibodies against this serovar were particularly detected in sow sera when the corresponding litter was *Leptospira* spp. PCR positive. Although this finding should be interpreted with caution given the low number of examined samples, this finding indicates a possible association between *Leptospira* serogroup Pomona and the clinical picture in our study, highlighting a relevant role of this serogroup in SMEDI. Indeed, serogroup Pomona is reported as clinically relevant and known as one of the most often detected ones in swine globally (Arent & Ellis, 2019; Fennestad & Borg-Petersen, 1966) which seems also be mirrored within the present study. In the acute phase of leptospirosis, broad cross-reactivity is observed, followed by relative serogroup specificity in convalescent-phase samples. This pattern reflects the detection by the microscopic agglutination test (MAT) of both IgM and IgG antibodies, as well as the presence of shared antigens among leptospires (Levett, 2001). Consequently, MAT can reliably indicate the infecting serovar only at later stages of infection, when high antibody titers are present. In cases of Pomona-associated abortions, most affected sows typically exhibit titers greater than 800 (Arent & Ellis, 2019) a finding that was consistent with all sow sera from PCR-positive litters in our study. In endemically infected herds with pig-adapted strains of Bratislava the frequency of sows with antibody titers of greater than 100 in the MAT is usually very low, although many sows will have titers of <100 (Arent & Ellis, 2019). From that perspective, serovars Pomona and Bratislava seem to be the relevant ones in the present study. High titers against other serovars in farm six might most probably be the result of vaccination against leptospires in this farm.

Referring to fetuses, due to hemolysis only a minority (23.0 %) of gained heart blood samples from fetuses with an estimated age > 70 days could be examined by MAT and the remaining sera were also of borderline quality, especially at the low dilution required, and contained a lot of free particles and/or cells, which made it difficult to assess specific agglutination with leptospires. Fetal heart blood of frozen fetuses therefore proved impractical for the detection of fetal antibodies against leptospires in this study. Besides the low quality of the heart blood, a short time to death after infection might also contribute to seronegative results.

A total of 14.1 % of fetuses tested positive for *Leptospira* spp. by PCR. In the present study, four fetuses per litter were sampled for diagnostic testing. This approach was guided by recommendations for abortion investigations, where sampling a limited number of fetuses has been considered sufficient to detect infectious agents (Althouse et al., 2019). Although SMEDI differs from classical abortion, and under the assumption that fetal death is directly caused by the pathogen all affected fetuses within a litter would be expected to test positive, evidence from abortion cases suggests that pathogen distribution within a litter may be heterogeneous and that not all fetuses necessarily test positive.

Therefore, the chosen sampling strategy represents a pragmatic balance between diagnostic sensitivity and practical feasibility. Nevertheless, the limited number of fetuses examined per litter should be considered when interpreting the results, as it may have contributed to an underestimation of the true number of Leptospira spp. positive litters.

Macerated fetuses showed the highest likelihood of detecting *Leptospira* DNA, whereas Ct values were not associated with fetal phenotype. This observation might be explainable due to bacterial decomposition of the fetuses due to bacterial infection (Lefebvre, 2015). Mummification was observed in eight of 20 *Leptospira* spp. PCR positive fetuses in the present study what goes in line with field observations in natural outbreaks of *Leptospira* spp. associated reproductive disorders (Fish et al., 1963) and the occurrence of mummified fetuses after inoculation of *Leptospira* serovar Pomona under controlled conditions (Jacobs et al., 2015). However, it remains unclear whether leptospires are the sole cause of the clinical outcome of the studied litters as co-infections might also contribute to pathogenesis. Data on the co-infections in our SMEDI data bank is published elsewhere (Eddicks et al., 2023) and point out to a relevant association between *Leptospira* spp. and porcine circovirus 2 in SMEDI litters. Thus, mummification could also be the consequence of additional viral infections such as ungulate protoparvovirus 1 or porcine circovirus 2, especially in SMEDI-cases. Numerically, the Lipl32-Gene PCR revealed more positive results compared to the 16SGene PCR and thus seems to be more suited for routine screening for *Leptospira* spp. DNA in clinical samples. However, kidney and liver samples were the most often positive ones and the ones with the lowest Ct-values in both PCRs, whereas stomach content and meconium samples revealed lower detection rate and higher Ct-values as well. These results seem to be in line with the pathogenesis of clinical leptospirosis in pigs. After the infection by penetration of mucosal surfaces or water softened skin, leptospires disseminate throughout the body within a bacteremia phase with replication in liver, kidney and lungs and other organs. Post septicemic leptospires persist in the tubules of the kidney nephron (Cianciolo & Mohr, 2016). These observations might happen analogue to fetuses after diaplacental infection and explain the laboratory diagnostic results. As stomach content is recommended as a specimen for bacterial infection of fetuses in terms of abortion (Althouse et al., 2019) it was analogously applied for diagnostics in SMEDI cases. Although it yielded numerically better results than meconium, the need to open fetal carcasses to obtain this sample makes it less suitable. In contrast, the easy and non-invasive collection of meconium (e.g., meconium swabs at the litter level) allows for larger sample sizes, which may compensate for its lower sensitivity.

## Conclusion

Within the study population, *Leptospira* spp. DNA was detected in a limited number of SMEDI-affected litters, and MAT titers against serovars Pomona and Autumnalis were only observed in sows associated with PCR-positive litters. Among the examined tissues, kidney samples were most frequently PCR-positive and showed the lowest Ct-values, indicating that they may represent the most suitable specimen for diagnostic screening. While meconium offers a non-invasive alternative that can be collected via rectal swabs, larger-scale studies are needed to further evaluate its diagnostic utility. Taken together, these findings suggest that an integrated diagnostic approach combining PCR, preferably performed on fetal kidney tissue, with serological testing (MAT) in the corresponding sows provides the most effective and practical strategy for detecting *Leptospira* spp. in SMEDI-associated reproductive disorders

## Ethical statement

The present examinations were approved by the internal ethical commission of the Veterinary Faculty of the LMU Munich (reference numbers 287- 06-10-2021 and 290-10-11-2021).

## Funding

This work was supported by MSD Animal Health, Intervet Deutschland GmbH.

## Declaration of generative AI and AI-assisted technologies in the writing process

The study and the preparation of the manuscript was done without the help of artificial intelligence.

## CRediT authorship contribution statement

**Matthias Eddicks:** Writing – original draft, Supervision, Project administration, Funding acquisition, Formal analysis, Data curation, Conceptualization. **Annika Seifert:** Writing – review & editing, Investigation, Formal analysis, Data curation. **Julia Gründl:** Writing – review & editing, Investigation, Formal analysis. **Katrin Strutzberg-Minder:** Writing – review & editing, Resources, Methodology, Investigation, Formal analysis. **Lina Eddicks:** Writing – review & editing, Resources, Methodology, Investigation. **Robert Tabeling:** Writing – review & editing, Project administration, Conceptualization. **Mathias Ritzmann:** Writing – review & editing, Supervision, Funding acquisition, Conceptualization.

## Declaration of competing interest

The authors declare the following financial interests/personal relationships which may be considered as potential competing interests: Matthias Eddicks reports financial support was provided by MSD Animal Health, Intervet Deutschland GmbH. Robert Tabeling reports a relationship with MSD Animal Health, Deutschland GmbH that includes: employment. If there are other authors, they declare that they have no known competing financial interests or personal relationships that could have appeared to influence the work reported in this paper.
